# Research on Real-Time Roundup and Dynamic Allocation Methods for Multi-Dynamic Target Unmanned Aerial Vehicles

**DOI:** 10.3390/s24206565

**Published:** 2024-10-12

**Authors:** Jinpeng Li, Ruixuan Wei, Qirui Zhang, Ruqiang Shi, Benqi Jiang

**Affiliations:** 1 Graduate College, Air Force Engineering University, Xi’an 710051, China; li_jinpeng118@163.com (J.L.);; 2 Aeronautics Engineering College, Air Force Engineering University, Xi’an 710038, China

**Keywords:** unmanned aerial vehicles, multi-target, real-time roundup, dynamic distribution

## Abstract

When multi-dynamic target UAVs escape, the uncertainty of the formation method and the external environment causes difficulties in rounding them up, so suitable solutions are needed to improve the roundup success rate. However, traditional methods can generally only enable the encirclement of a single target, and when the target is scattered and escaping, this will lead to encirclement failure due to the inability to sufficiently allocate UAVs for encirclement. Therefore, in this paper, a real-time roundup and dynamic allocation algorithm for multiple dynamic targets is proposed. A real-time dynamic obstacle avoidance model is established for the roundup problem, drawing on the artificial potential field function. For the escape problem of the rounding process, an optimal rounding allocation strategy is established by drawing on the linear matching method. The algorithm in this paper simulates the UAV in different obstacle environments to round up dynamic targets with different escape methods. The results show that the algorithm is able to achieve the rounding up of multiple dynamic targets in a UAV and obstacle scenario with random initial positions, and the task UAV, which is able to avoid obstacles, can be used in other algorithms for real-time rounding up and dynamic allocation. The results show that the algorithm is able to achieve the rounding up of multi-dynamic targets in scenarios with a random number of UAVs and obstacles with random locations. It results in a 50% increase in the rounding efficiency and a 10-fold improvement in the formation success rate. And the mission UAV is able to avoid obstacles, which can be used in other algorithms for real-time roundup and dynamic allocation.

## 1. Introduction

Cooperative UAV roundup technologies are increasingly vital for modern defense and security [1,2,3]. With the complexity and variability of the environment, a single UAV is no longer sufficient to meet the needs of complex tasks, such as emergency rescue, traffic control, environmental monitoring and other tasks, which often require a variety of models, multiple sorties, and multi-layer gradients of UAVs to work together to complete the corresponding tasks [4,5,6,7,8]. In the process of multi-UAV cooperation, how the UAV itself is localized in the group and how it interacts with the external environment is one of the key technologies studied [9,10,11,12].

As early as 1939, social psychologist Lewin conducted an in-depth study on the Cooperation of Humans (CoH). He pointed out that human cooperation is a complex group behavior, subject to the joint actions of each individual within the group and the external environment, and based on this, he proposed a group cooperation model [13,14]. The CoH model reveals the general principles of human collaborative behavior to a certain extent; however, it is a passive cooperation model without a clear goal. Groups do not engage with each other in meaningless interactions, but collaborate purposefully, i.e., goal-oriented group collaboration. In a collaborative task, each person should have their own task, and they should act independently of each other to accomplish individual tasks as well as work in parallel to accomplish group tasks. For this reason, based on this method, this paper further considers the constraints between the roundup task and the UAV cluster, and based on the sharing of environmental information perceived by the distributed UAVs, converges the multiple targets to form the group’s collaborative strategy and the individual’s control scheme, and presents research on the real-time roundup and dynamic allocation method of multi-dynamic target UAVs, which can solve the problem of multiple UAV roundups [15,16,17].

In terms of group and individual UAV cooperative control, after the decision-making strategy is generated, it is not only necessary to consider the control of the group as a whole oriented to the purpose of rounding up, but also the individual control of each UAV [18,19]. At this point, it is necessary to consider the state, position, and other information of the UAVs, and to make targeted adjustments to the control strategy, ensuring that the group of UAVs can collaborate to complete the rounding up of the target [20,21,22].

The above literature provides some results in the area of dynamic target fencing and obstacle avoidance. However, most of these traditional methods can only encircle a single target. This means that if there are multiple target UAVs with scattered escapes, these methods will fall into a local optimum due to the inability to determine the target to be rounded up, leading to roundup failure. Furthermore, there will be inappropriate allocation of roundups, resulting in an insufficient number of roundups, too great of a distance distance, etc., causing the partial or total failure of the roundup mission.

Traditional obstacle avoidance methods usually deal with the problem of a single UAV arriving at a single target, and global information about environmental obstacles is often obtained before planning, which makes it impossible to avoid dynamic obstacles; the artificial potential field method is capable of dealing with dynamic obstacle problems, but it is prone to falling into local optima. Researchers have improved the above methods and proposed new algorithms that can achieve obstacle avoidance. Due to the emergence of multi-objective missions, it is impossible or difficult for a single UAV to complete the mission. This requires the use of multiple UAVs to deal with the multi-target problem, and cooperative obstacle avoidance by multiple UAVs is the key to solving this problem. In traditional methods, intelligent algorithms such as genetic algorithms and swarm algorithms are not suitable for solving this problem due to the increase in the number of UAVs, which leads to an exponential and dramatic increase in the amount of computation. The artificial potential field method is suitable for this problem, but involves an interaction between the mission UAV and the obstacles and the target UAV to avoid obstacles, which requires the algorithm to be improved to balance each parameter to obtain a suitable obstacle avoidance method. In this paper, based on the artificial potential field method, a dynamic obstacle avoidance model is established using boundary thresholding, which can maintain the formation and distance of the roundup while achieving obstacle avoidance.

In summary, the main contributions of this paper are as follows:

(1) A real-time dynamic obstacle avoidance model is designed based on an artificial potential field function, in which all mission UAVs are able to avoid obstacles relatively smoothly under continuously applied perturbations. Moreover, there is also a potential field constraint between UAVs in producing a certain roundup formation.
(2) A multi-dynamic target allocation scheme is designed to converge the environmental information perceived by multiple targets to form a collaborative strategy for the group and a control scheme for the individual based on further consideration of the group UAV constraints. The optimal allocation strategy is derived by establishing an evaluation index function, linearly matching from the perspective of the target UAVs, and combining the total cost of all the mission UAVs.
(3) The path of the roundup process is smoothed and optimized. The planned paths of all the UAVs are jittery due to the setup of continuous interference. The paths of all the UAVs are smoothed by means of Bessel curves to obtain smooth and stable feasible roundup flight paths.

## 2. Preliminaries

### 2.1. Problem Model

The UAV maintains a fixed altitude during level flight for roundup operations, and the kinematic equation of the UAV is

(1) $$\left[\begin{array}{c} \dot{x}\\ \dot{y}\\ \dot{\theta } \end{array}\right]=\left[\begin{array}{cc} \cos\theta & 0\\ \sin\theta & 0\\ 0 & 1 \end{array}\right]\cdot \left[\begin{array}{c} \nu \\ \omega \end{array}\right]$$

where x,y is the current position of the UAV, θ is the heading angle, v is the current linear velocity, and ω is the current angular velocity. Accounting for perturbations $\theta_{dis}$, the UAV’s motion equation is rewritten as

(2) $$\left[\begin{array}{c} \dot{x}\\ \dot{y}\\ \dot{\theta } \end{array}\right]=\left[\begin{array}{cc} \cos(\theta +\theta_{dis}) & 0\\ \sin(\theta +\theta_{dis}) & 0\\ 0 & 1 \end{array}\right]\cdot \left[\begin{array}{c} \nu \\ \omega \end{array}\right]$$

There exists a minimum turning radius $R_{\min}$ for the UAV and the yaw angle constraint is satisfied $\left|\omega \right|\leq v/R_{\min}$.

Based on the position $\left(x_{k},y_{k}\right)$ of the mission UAV and the position $\left(x_{g},y_{g}\right)$ of the target UAV, it is possible to calculate the angle of the UAV rounding as

(3) $$\theta_{k}=\arctan((y_{g}-y_{k})/(x_{g}-x_{k}))$$

**Figure 1:**
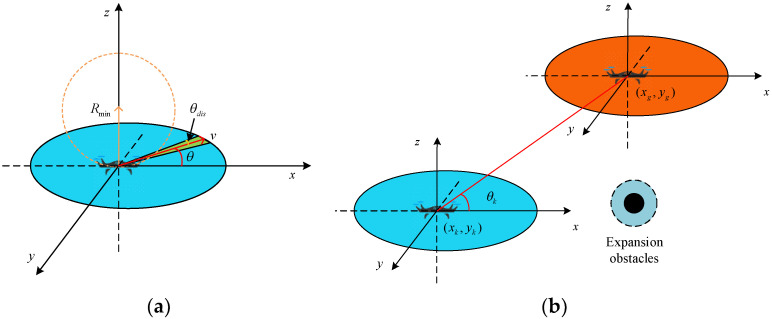
Three-dimensional view of the UAV geometry relationship. (**a**) Geometric modeling of UAVs; (**b**) positional relationships between UAVs.

There is a safe distance between the UAV and the obstacle, and the safe distance is determined by expanding the obstacle. See Figure 1.

### 2.2. Artificial Potential Field

The basic idea of the traditional artificial potential field is to construct the environmental attraction potential field and repulsion potential field for the environmental information in which the controlled object is located, to guide the controlled object to avoid obstacles and arrive at the target point.

According to the concept of potential field in physics, the obstacle body is constructed to produce repulsive force potential field. When the UAV enters the scope of action of the repulsive force potential field, it begins to be subjected to repulsive force. The smaller the distance between the UAV and the obstacle, the greater the repulsive force, driving the UAV away from the obstacle. Constructing the target point to produce an attractive potential field to the UAV, the scope of action covers the entire map. The UAV will always be attracted by the target point after take-off, and the strength of the action is proportional to the Euclidean distance between the two, guiding the UAV to fly towards the target point. The artificial potential field function established by the UAV is

(4) $$U_{rep}(x)=\left\{ \begin{array}{l} \frac{1}{2}\alpha {\left(\frac{1}{\left\|x_{o}-x\right\|}-\frac{1}{x_{r}}\right)}^{2},\left\|x_{o}-x\right\|\leq x_{r}\\ 0,\left\|x_{o}-x\right\|>x_{r} \end{array}\right.$$

(5) $$U_{att}(x)=\frac{1}{2}k{\left\|x-x_{g}\right\|}^{2}$$

(6) $$U_{all}(x)=U_{rep}(x)+U_{att}(x)$$

In Equations (4)–(6), $U_{rep}(x)$ is the repulsive potential function of the obstacle to the UAV; $U_{att}(x)$ is the attractive potential function of the target point to the UAV; $U_{all}(x)$ is the combined potential function of the target point to the UAV; $x_{o}$ is the position of the obstacle; x is the current position of the UAV; $x_{g}$ is the position of the target point; $x_{r}$ is the range of the repulsive potential field of the obstacle’s influence; α is the gain coefficient of the repulsive potential field; and k is the gain coefficient of the attractive potential field.

The repulsive force and the gravitational force on the UAV are obtained sequentially by finding the negative gradient for $U_{rep}(x)$ and $U_{att}(x)$, respectively, and the direction of the combined force of the two is the direction of the UAV’s motion. The negative gradient of the potential function is obtained as the force function:

(7) $$F_{rep}(x)=-\nabla U_{rep}(x)=\left\{ \begin{array}{l} \alpha (\frac{1}{\left\|x_{o}-x\right\|}-\frac{1}{x_{r}})\frac{1}{{\left\|x_{o}-x\right\|}^{2}},\left\|x_{o}-x\right\|\leq x_{r}\\ 0,\left\|x_{o}-x\right\|>x_{r} \end{array}\right.$$

(8) $$F_{att}(x)=-\nabla U_{att}(x)=-k\left\|x-x_{g}\right\|$$

(9) $$F_{all}(x)=\sum F_{rep}(x)+F_{att}(x)$$

In Equations (7)–(9), $F_{rep}(x)$ is the repulsive force of the obstacle on the UAV; $F_{att}(x)$ is the attractive force of the target point on the UAV; and $F_{all}(x)$ is the combined force on the UAV, which consists of the superposition of the repulsive force of all obstacles in the obstacle’s influence range and the attractive force of the target point.

## 3. Design of Algorithm

In traditional target roundup algorithms, when task UAVs encounter areas with numerous obstacles, because the target UAV and the obstacles are in the same straight line, as the task UAV approaches the target UAV, the distance from the obstacles decreases, and at this time, the task UAV is subjected to obstacle repulsion increase, which in turn will force the task UAV far away from the target UAV. The mission UAV cannot penetrate the obstacle area, resulting in failed target roundup.

**Figure 2:**
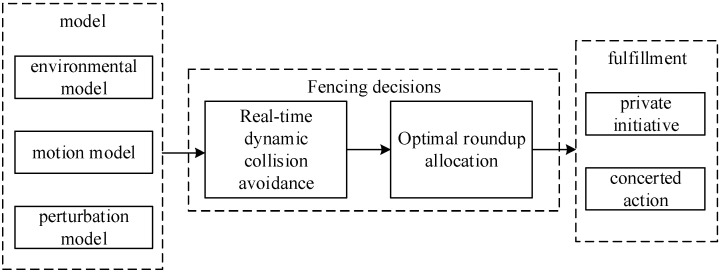
Block diagram of the roundup program.

To address this problem, a dynamic allocation scheme for multi-dynamic target roundup is designed. According to the escape mode in which the target UAV is located, the task UAV is assigned to implement the roundup in real time. The block diagram of the roundup is shown in Figure 2.

**Figure 3:**
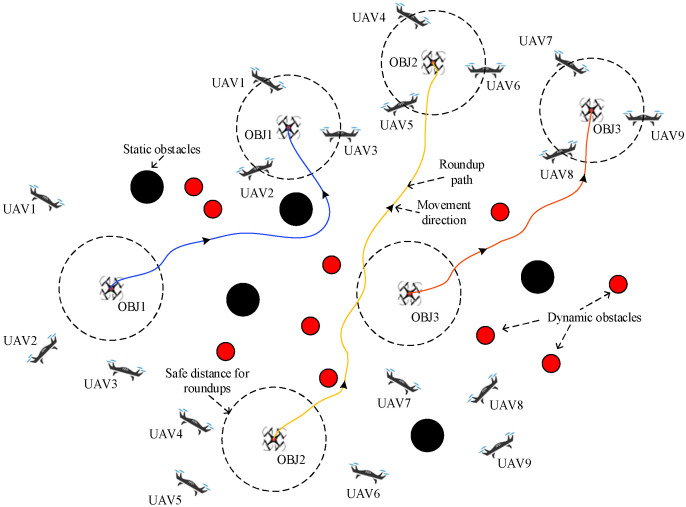
Schematic diagram of the effect of roundup.

The roundup scheme assumes the existence of multiple mission UAVs and multiple target UAVs, and assigns a corresponding number of mission UAVs to implement roundup according to the escape mode of the target UAVs. As can be seen from Figure 2, the scheme has two main components: the real-time dynamic obstacle avoidance algorithm and the optimal roundup allocation strategy. The real-time dynamic obstacle avoidance algorithm is used for flight path planning, and when there are obstacles near the task UAVs, the corresponding potential field is derived based on the distance calculation, which determines the next action to be executed by the task UAVs. Eventually, a safe distance is maintained with the target UAV when it arrives near the roundup target UAV. The optimal roundup allocation algorithm allocates a reasonable roundup scheme, allocates a certain number of mission UAVs for roundup according to the escape formation and number of target UAVs, and forms a roundup formation to ensure the roundup effect. The roundup scheme combines the above two parts to realize the real-time roundup of dynamic targets and the dynamic allocation of roundup UAVs. See Figure 3.

### 3.1. Real-Time Dynamic Obstacle Avoidance Based on Boundary Thresholding

Each individual UAV performs an individual behavior based on their respective perceived information I(x,y,z), and is able to avoid dynamic and static obstacles in the environment, at the same time being able to pursue the target UAV. However, the independent individuals do not perceive the global information as a whole, and the global perception information WI(x,y) is formed by fusing the distributed independent individual information.

Assuming that there are $M_{k}$ remaining UAVs within the perception range of the mission UAV k, the relative distance matrix $D_{k}$ between the $M_{k}$ UAVs and UAV k can be obtained separately:

(10) $$D_{k}={\left[D\mathrm{is}\left(u_{k},u_{1}\right),D\mathrm{is}\left(u_{k},u_{2}\right),\ldots ,D\mathrm{is}\left(u_{k},u_{M_{k}}\right)\right]}^{T}$$

(11) $$D\mathrm{is}\left(u_{k},u_{M_{k}}\right)=\sqrt{{\left(x_{u_{k}}-x_{u_{M_{k}}}\right)}^{2}+{\left(y_{u_{k}}-y_{u_{M_{k}}}\right)}^{2}}$$

where $D_{k}$ denotes the relative distance matrix between the $M_{k}$ UAVs and UAV k. Each element $D\mathrm{is}\left(u_{k},u_{M_{k}}\right)$ in its distance matrix is calculated as shown in the above equation.

In order to ensure the safety of UAV interactions, the UAVs need to have anti-collision capabilities. The UAVs need to maintain a safe distance between themselves within their sensing range, and only need to maintain a safe distance with other UAVs closest to UAV k itself.

Assume that for any UAV $q\in M_{k}$, $D\mathrm{is}\left(u_{k},u_{q}\right)=\min\left(D_{k}\right)$, min are the minimum values in the matrix. It can be derived that the remaining UAV in the closest sensing range to UAV k is q, and the force ${\vec{F}}_{k}^{q}$ it receives from UAV q is

(12) $${\vec{F}}_{k}^{q}=\left\{ \begin{array}{ll} -\mu_{1}{\vec{d}}_{kq}\cdot \frac{\left\|{\vec{d}}_{kq}\right\|-R_{1}}{{\left\|{\vec{d}}_{kq}\right\|}^{\lambda +1}} & \;,\left\|{\vec{d}}_{kq}\right\|\leq R_{1}\\ \;\;\;\;0 & \;,R_{1}<\left\|{\vec{d}}_{kq}\right\|\leq R_{2}\\ \mu_{1}{\vec{d}}_{kq}\cdot \frac{\left\|{\vec{d}}_{kq}\right\|-R_{2}}{{\left\|{\vec{d}}_{kq}\right\|}^{\lambda +1}} & \;,R_{2}<\left\|{\vec{d}}_{kq}\right\|\leq R_{3} \end{array}\right.$$

where $\mu_{1}$ is a constant coefficient, λ is an exponential constant coefficient, and $R_{1}$, $R_{2}$ and $R_{3}$ denote the repulsive region boundary value, equilibrium region boundary value, and gravitational region boundary value of the UAV, respectively.

In order to ensure the collaborative following of UAVs, it is necessary that the UAVs are not too far away from each other in the sensing range, thus making it easier to form tight formations. At this point, it is only necessary for UAV k to maintain a formation distance with the UAV furthest away from itself.

Assume that for any UAV $p\in M_{k}$, there are $D\mathrm{is}\left(u_{k},u_{p}\right)=\max\left(D_{k}\right)$, max as the maximum value in the fetch matrix. It can be derived that the farthest remaining UAV within the perception range of UAV k is p, and the force it receives from UAV p is

(13) $${\vec{F}}_{k}^{p}=\left\{ \begin{array}{ll} -\mu_{2}{\vec{d}}_{kp}\cdot \frac{\left\|{\vec{d}}_{kp}\right\|-R_{1}}{{\left\|{\vec{d}}_{kp}\right\|}^{\lambda +1}} & \;,\left\|{\vec{d}}_{kp}\right\|\leq R_{1}\\ \;\;\;\;0 & \;,R_{1}<\left\|{\vec{d}}_{kp}\right\|\leq R_{2}\\ \mu_{2}{\vec{d}}_{kp}\cdot \frac{\left\|{\vec{d}}_{kp}\right\|-R_{2}}{{\left\|{\vec{d}}_{kp}\right\|}^{\lambda +1}} & \;,R_{2}<\left\|{\vec{d}}_{kp}\right\|\leq R_{3} \end{array}\right.$$

where $\mu_{2}$ is a constant coefficient, λ is an exponential constant coefficient, and $R_{1}$, $R_{2}$ and $R_{3}$ denote the repulsive region boundary value, the equilibrium region boundary value, and the gravitational region boundary value of the UAV, respectively.

Then, the combined force ${\vec{F}}_{k}$ on UAV k is the sum of the nearest force ${\vec{F}}_{k}^{q}$ and the farthest force ${\vec{F}}_{k}^{p}$, i.e., ${\vec{F}}_{k}={\vec{F}}_{k}^{q}+{\vec{F}}_{k}^{p}$.

The traditional artificial potential field method easily falls into local minima, which leads to planning failure. In this paper, the improved artificial potential field function not only ensures the safe distance of the UAV’s flight, but also ensures the stable following of the formation. The proposed method sets the gravitational region with the equilibrium region and the repulsion region, and the UAV converges to the equilibrium region due to the gravitational force and the repulsion force. In the equilibrium region, the UAV is able to move steadily towards the target point. When leaving the equilibrium region, it is again subjected to gravitational and repulsive forces, which keep the UAV in the equilibrium region between the repulsive and gravitational regions, i.e., between $R_{2}$ and $R_{3}$.

For the obstacle in the sensory information of UAV k, assuming that the obstacle point $\left(x_{op},y_{op}\right)$ is the nearest obstacle collision avoidance point, UAV k receives the repulsive force $F_{k}^{o}$ on it from this obstacle point as

(14) $${\vec{F}}_{k}^{o}=\beta \cdot \frac{{\left(D_{\max}-d_{ko}\right)}^{2}}{d_{ko}^{3}}\frac{{\vec{d}}_{ko}}{\left\|{\vec{d}}_{ko}\right\|}=\beta \cdot {\vec{d}}_{ko}\frac{{\left(D_{\max}-d_{ko}\right)}^{2}}{d_{ko}^{4}}$$

where β is the obstacle point repulsion coefficient, and $D_{\max}$ is the farthest obstacle distance that the UAV can sense, i.e., the maximum distance of obstacle point repulsion.

### 3.2. Identify Target Escape Modes

There are three types of escape methods for target UAVs, namely, dispersed escape, formation escape, and group escape. The escape mode can be judged in real time according to the distance between the target UAVs.

Assuming that there are M target UAVs within the sensing range of target UAV g, the relative distance matrix D between M UAVs and UAV g can be obtained separately:

(15) $$D_{g}={\left[D\mathrm{is}\left(u_{g},u_{1}\right),D\mathrm{is}\left(u_{g},u_{2}\right),\ldots ,D\mathrm{is}\left(u_{g},u_{M_{g}}\right)\right]}^{T}$$

(16) $$D\mathrm{is}\left(u_{g},u_{M_{g}}\right)=\sqrt{{\left(x_{u_{g}}-x_{u_{M_{g}}}\right)}^{2}+{\left(y_{u_{g}}-y_{u_{Mg}}\right)}^{2}}$$

where $D_{g}$ denotes the relative distance matrix between $M_{g}$ UAVs and UAV g. Each element $Dis\left(u_{g},u_{M_{g}}\right)$ in its distance matrix is calculated as shown in the above equation.

When the distance $D_{k}>R$ between the target UAVs displays dispersed escape, when the distance $D_{k}<R$ between the target UAVs displays formation escape, and when the distance between the target UAVs is both greater than R and less than R, it this is termed group escape.

The main purpose of a mission UAV is to round up a target UAV. To achieve the roundup, the mission UAV should fly towards the location of the rounded-up target UAV. The coordinates of the centre of mass of the mission UAV are defined as follows:

(17) $$x_{s}=\frac{\sum_{k=1}^{n}x_{k}}{n},y_{s}=\frac{\sum_{k=1}^{n}y_{k}}{n}$$

where n is the total number of task UAVs, and $x_{k}$ and $y_{k}$ are the horizontal and slave coordinates of the task UAVs, respectively.

Mission UAVs are able to fuse to form global sensory information through information exchange between them. The target UAV can be sensed by one or more mission UAVs in the surrounding environment. Let the coordinates of the target UAV be rounded up to $\left(x_{f},y_{f}\right)$, make the mission UAV fly towards the target UAV to be rounded up, and construct the distance function between them as

(18) $${\vec{F}}_{f}^{s}=\frac{{\vec{d}}_{sf}}{\left\|{\vec{d}}_{sf}\right\|}\cdot \left[\omega_{1}\left(d_{sf}-D_{\min}^{sf}\right)+\omega_{2}\cdot \left(D_{\max}^{sf}-d_{sf}\right)+\omega_{3}\cdot d_{sf}\right]$$

where $d_{sf}$ is the distance between the center of mass of the mission UAV swarm and the target to be rounded up, $\frac{{\vec{d}}_{sf}}{\left\|{\vec{d}}_{sf}\right\|}$ is the direction that guides the mission UAV swarm towards the target UAV to be rounded up, $D_{\min}^{sf}$ is the distance from the closest UAV in the mission UAV swarm to the target UAV to be rounded up, $D_{\max}^{sf}$ is the distance from the furthest UAV in the mission UAV swarm to the target UAV to be rounded up, $\omega_{1}$, $\omega_{2}$, and $\omega_{3}$ are weight constants, respectively.

### 3.3. Assignment Roundup Distribution Strategy

The linear allocation method is used to obtain the roundup program with the smallest overall cost. Firstly, the interactive behavioral relationships between the mission UAV and the target UAV and the environment are analyzed. Then, the mapping relationship network between all the UAVs is obtained. Then, the evaluation index function of the UAVs is constructed. Finally, the roundup scheme with the lowest total cost is calculated.

The set of individual behaviors $S_{u}$ is made by each mission UAV $u_{k}$ independently sensing information on the environment, and the set of global behaviours $S_{a}$ generated by the mission UAVs interacting with the target UAVs, using the UAV correlation judgement function rel to derive the mapping network NET formed between the set of individual behaviors $S_{u}$ and the set of global behaviors $S_{a}$:

(19) $$rel\left(u_{k},S_{u},S_{a}\right)=Hof_{u_{k}}\left(S_{u}\right)\mapsto S_{a}$$

(20) $$\mathrm{NET}:\left\{net\left(s_{i},s_{j}\right)\right\}\;,\;if\;rel\left(u_{k},S_{u},S_{a}\right)\;,\;s_{i}\in S_{u}\;,\;s_{j}\in S_{a}$$

where $Hof_{u_{k}}\left(\cdot \right)$ denotes the UAVs within the perception range of the task UAV $u_{k}$, and $net\left(s_{i},s_{j}\right)$ denotes the behavioral mappings formed between UAV $u_{k}$ and the UAVs within the perception range, and the mapping network NET can be formed by integrating all the behavioral mappings $net\left(s_{i},s_{j}\right)\;,\;s_{i}\in S_{u}\;,\;s_{j}\in S_{a}$.

After the mapping network NET is constructed, the completeness of the network needs to be evaluated, and the evaluation network W is constructed as a function of all the behavioral mappings in the behavioral network involving between the mission UAV and the target UAV:

(21) $$W=\left\{ \begin{array}{l} W_{1}:f\left(\mathrm{NET},rel\left(u_{1},S_{u},S_{a}\right)\right)\\ W_{2}:f\left(\mathrm{NET},rel\left(u_{2},S_{u},S_{a}\right)\right)\\ \vdots \\ W_{n}:f\left(\mathrm{NET},rel\left(u_{n},S_{u},S_{a}\right)\right) \end{array}\right.$$

where $W_{k}:f\left(\mathrm{NET},rel\left(u_{k},S_{u},S_{a}\right)\right)$ is defined as the evaluation metric function $W_{k}$ for UAV $u_{k}$:

(22) $$W_{k}:f\left(\mathrm{NET},rel\left(u_{k},S_{u},S_{a}\right)\right)≜\exists net\left(s_{k},\cdot \right)\in \mathrm{NET},rel\left(u_{k},S_{u},S_{a}\right)⊗\mathrm{NET}=\max\left(rel\left(\mathrm{NET},S_{u},S_{a}\right)\right)$$

where ≜ denotes “equivalent to”, ∃ denotes existence, ∈ denotes belonging, ∈ is the cross-multiplication function, and max(⋅) is the maximization function.

At this point, the optimal allocation of *n* mission UAVs to round up m target UAVs can be achieved by minimizing the total cost after allocation.

(23) $$\underset{D}{\min}\sum_{ij}D_{ij}{W_{k}}^{'}$$

(24) $$\sum_{i}D_{ij}=1,j=1,2,\ldots ,n$$

(25) $$\sum_{j}D_{ij}=k,i=1,2,\ldots ,m$$

(26) $$D_{ij}\in \left\{0,1\right\},i,j=1,2,\ldots ,m,\ldots n$$

where $W_{k}$ is the evaluation metrics function and $W_{k}\in W$. $D_{ij}\in \left\{0,1\right\}$ is an indication of whether or not to assign. Here, $D_{ij}=1$ indicates that the i-th mission UAV is assigned to the j-th target UAV and $D_{ij}=0$ indicates that the i-th mission UAV is not assigned to the j-th target UAV. Equation (24) indicates that the task UAV selects only one target UAV in the current state. Equation (25) indicates that the target UAV in the current state is simultaneously rounded up by k mission UAVs. Since the roundup mission is performed, the number of mission UAVs generally needs to be larger than the number of target UAVs, so there are m<n. Here, m is the total number of target UAVs and n is the total number of mission UAVs.

**Figure 4:**
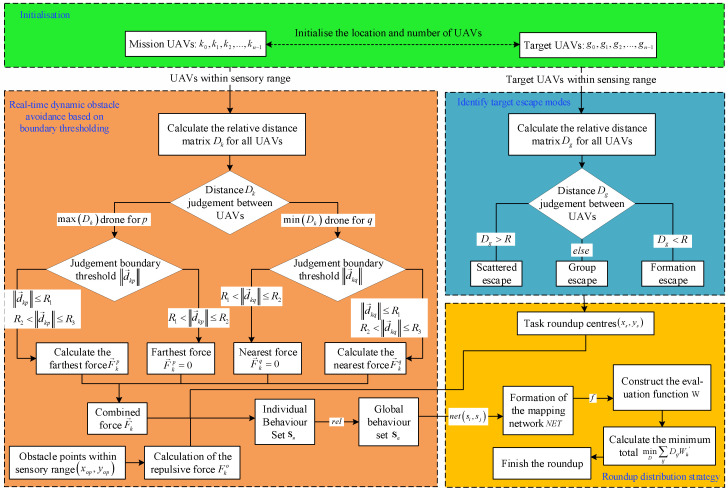
Flowchart of the roundup allocation strategy.

In summary, the flowchart of the multi-UAV collaborative roundup distribution method is as follows. See Figure 4.

## 4. Simulation and Analysis of Results

### 4.1. Simulation Settings

Typically, the initial positions of the mission and target UAVs vary based on actual scenarios. Therefore, a random initial position is more reasonable, and the algorithm sets the initial positions of all the UAVs to be taken randomly between [20, 180]. And static and dynamic obstacles are also set within this interval. Accordingly, the quantities, radii and speeds are shown in Table 1.

The number of mission UAVs was set to nine, aiming to verify the effectiveness of medium-scale mission UAV roundups. The number of target UAVs is set to three, which is intended to verify the escape of small-scale targets. In practice, the number of UAVs can be increased or decreased according to the specific situation. In the simulation, the radii of the UAVs are all set to 1 m to ensure a certain flight safety distance. The number of static obstacles is set to five with a radius of 10 m, which is used to simulate the presence of fixed obstacles. The number of dynamic obstacles was set to 10, with a radius set to 5 m, to simulate moving objects. The speed of the UAV and the obstacles were set according to the actual situation.

To enhance the environmental complexity, the trajectories of dynamic obstacles are randomly adjusted based on their current positions. In this case, Δx is added in the direction of the x-axis and Δy is added in the direction of y-axis. And Δx∈(−10,20),Δy∈(−10,20), the positivity and negativity of the taken values ensures that the dynamic obstacle can move in all directions. Secondly, setting positive values greater than negative values provides better assurance that the obstacle is moving roughly in the direction of the first quadrant.

To ensure the physical implementation of the UAS and the repeatability of the experiments, the key performance indicators for each component are shown in Table 2 below. The parameter settings and data for all components in the table are physically implementable and repeatable.

Table 3 shows the setting support and data table for the parameters. In the table, the weight constants, $\omega_{1}$, $\omega_{2}$ and $\omega_{3}$, can be adjusted according to the actual situation. In Table 3, $\omega_{1}=\omega_{2}=\omega_{3}=1$ indicates that the three distances have the same effect on the roundup effect. When the value of $\omega_{1}$ is appropriately boosted, the distance $d_{sf}$ between the centre of mass of the mission UAV population and the target to be rounded up is valued more, bringing the UAV population closer to the target. When the value of $\omega_{2}$ is appropriately boosted, more weight is given to the distance $D_{\min}^{sf}$ of the UAV closest to the target UAV to be rounded up in the mission UAV swarm, allowing the UAV swarm to respond to the roundup more quickly. When the value of $\omega_{3}$ is appropriately boosted, more weight is given to the distance $D_{\max}^{sf}$ of the UAV in the mission UAV swarm that is furthest away from the target UAV to be rounded up, to avoid the target escaping.

The action simulation experiment is divided into three groups according to the escape mode of the roundup target UAVs. They are, respectively, three target UAVs displaying dispersed escape, three target UAVs displaying formation escape, and three target UAVs displaying group escape. In each group of simulation experiments, for the sake of the fairness of the simulation experiments, the above four environment maps are simulated separately for the case of no obstacle, the case of static obstacle only, the case of dynamic obstacle only, and the case of both static obstacle and dynamic obstacle.

### 4.2. Result Analysis

(1) Scattered escape: nine mission UAVs rounding up three dynamic targets

**Figure 5:**
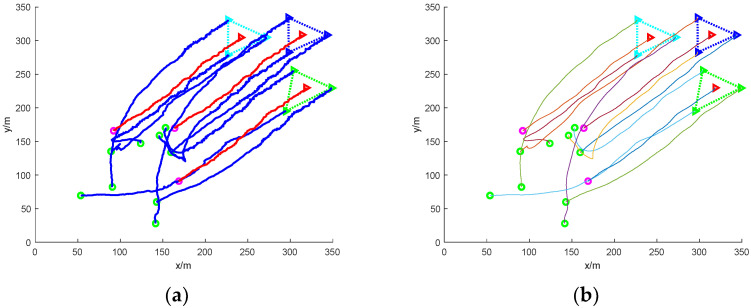
Nine UAVs rounding up three dispersed dynamic targets without obstacles. (**a**) The roundup process; (**b**) smoothing of the roundup process.

Figure 5 shows a simulation of nine mission UAVs rounding up three target UAVs under accessibility. In the figure, the green circle indicates the initial position of the nine task UAVs; the magenta circle indicates the initial position of the three target UAVs; the green, blue, and cyan triangles indicate the current position of the UAVs that perform the roundup task and the three UAVs of the same colour round up the same target; the red triangle indicates the current position of the target UAVs; and the curves in the figure are the UAV’s flight trajectories.

In the figure, it can be seen that due to the dispersed escape of the three target UAVs, the task UAVs need to carry out a reasonable allocation of the task UAVs if they want to carry out the roundup, so, starting from a random initial position, based on the calculation of the distance between the individual task UAVs and the individual target UAVs, linear matching is carried out, and the closest set of solutions is obtained, and the task UAVs soon form three teams, which, respectively, encircle the three dispersed escaping target UAVs and finally complete the roundup.

Since the position interference quantity is applied to the path, the flight trajectory of the UAV in Figure 5a is a nonlinear zigzag path, so the Bessel curve is used to smooth the path, and a stable and smooth path is obtained, as shown in Figure 5b. 

**Figure 6:**
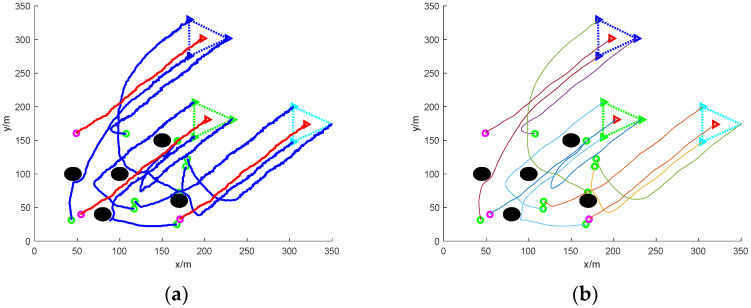
Nine UAVs rounding up three dispersed dynamic targets under static obstacles. (**a**) The roundup process. (**b**) Smoothing of the roundup process.

Figure 6 shows the simulation of nine UAVs rounding up three dispersed fleeing dynamic targets under static obstacles. Five static obstacles with a radius size of 10 m are added to Figure 6, and the static obstacles are represented by black circular patterns.

In the figure, it can be seen that at the initial moment, the mission UAV and the target UAV are randomly distributed in the environment map. The target UAV starts to disperse and escape, and the task UAV needs to sense the static obstacles in the environment and avoid collisions while linearly matching the target UAV. The final simulation results show that the task UAV is able to round up the scattered target UAVs and the trapping path is smoothed with Bessel curves.

**Figure 7:**
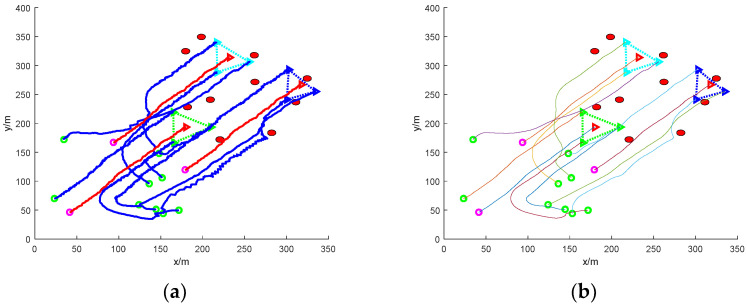
Nine UAVs rounding up three dispersed dynamic targets under dynamic obstacles. (**a**) The roundup process. (**b**) Smoothing of the roundup process.

Figure 7 shows the simulation of nine mission UAVs rounding up three dispersed fleeing target UAVs under dynamic obstacles. Ten dynamic obstacles with a radius size of 5 m are added to Figure 5, and the dynamic obstacles are represented by red circular patterns.

In the figure, the addition of dynamic obstacles makes it necessary for the mission UAV to sense the environmental information online to change the flight direction to avoid the dynamic obstacles during the flight. The UAV moves with the gradient direction of the ensemble potential field and takes the assigned target UAV as the centre of the encirclement, respectively, and finally succeeds in encircling the dispersed escaping target UAV.

Figure 6 shows the simulation of nine UAVs rounding up three dispersed dynamic targets under mixed obstacles. It combines the static obstacle case of Figure 4 and the dynamic obstacle case of Figure 5.

The mission process is nine mission UAVs randomly distributed in the environment map to round up three dispersed escaping target UAVs. The environment map includes 5 static obstacles drawn in black circles and 10 dynamic obstacles drawn in red circles. The static obstacles have a radius of 10 m and the dynamic obstacles have a radius of 5 m. From the simulation map, it can be seen that the UAV completes the rounding up of the dispersed fleeing dynamic target with mixed obstacles and smooths the path of rounding up.

**Figure 8:**
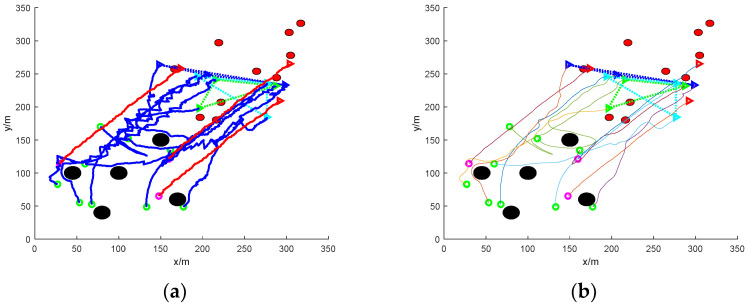

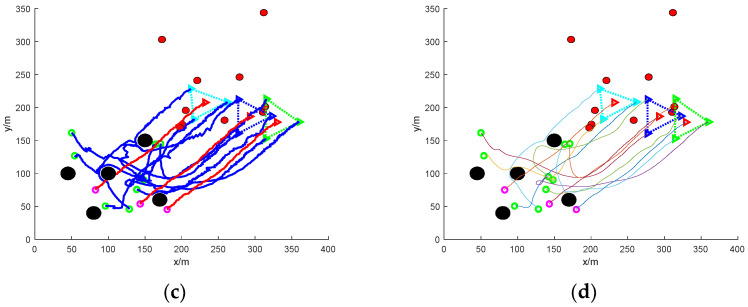
Nine UAVs rounding up three dispersed dynamic targets under mixed obstacles. (**a**) Traditional methods of roundup process. (**b**) Smooth traditional roundup process. (**c**) The roundup process of this paper’s method. (**d**) Smoothing of the roundup process of the method in this paper.

The traditional method fails in the process of rounding up dispersed fleeing targets due to the presence of distribution problems. As can be seen in Figure 8a, there are more twists and turns in the flight path of the UAV. Figure 8b smooths the roundup process and the resulting path is somewhat improved. The traditional method can only select target UAVs for rounding up by means of distance cost, and when the mission UAVs are close to the distance cost of multiple target UAVs, it will be impossible to determine the specific target for rounding up. In turn, the formation of the mission UAVs will also fall into this local optimum, leading to the failure of roundup.

Figure 8c shows the rounding process of this paper’s method under mixed obstacles, and Figure 8d shows the smoothing of the rounding process. In the figure, it can be clearly seen that the path of this paper’s method is smoother compared to the traditional roundup method roundup. The method in this paper can successfully round up the dispersed escaping targets.

(2) Formation escape: nine UAVs working together to round up three formation targets

**Figure 9:**
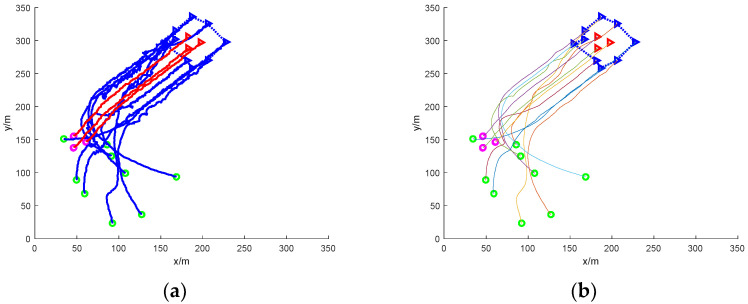
Nine UAVs rounding up three formation dynamic targets without obstacles. (**a**) The roundup process. (**b**) Smoothing of the roundup process.

Figure 9 shows the simulation of nine UAVs rounding up three formation dynamic targets without obstacles. Again, the blue triangles in the figure indicate the mission UAVs and the three triangles indicate the target UAVs. It can be found that the mission UAVs quickly form a roundup of the target UAVs in an obstacle-free environment. The overall rounding process is relatively stable due to the absence of obstacles, followed by smoothing of the path using Bessel curves.

**Figure 10:**
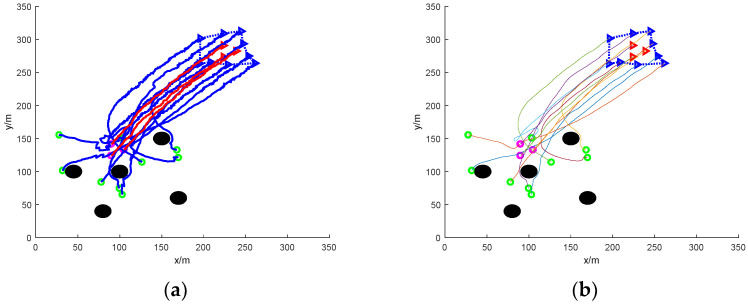
Nine UAVs under static obstacles to round up three formation dynamic targets. (**a**) The roundup process. (**b**) Smoothing of the roundup process.

Figure 10 shows the simulation of nine UAVs surrounding three formation dynamic targets under static obstacles. In the figure, the mission UAVs initially distributed in the area surrounded by static obstacles need to avoid the obstacles while pursuing the target UAVs. The task UAVs are able to avoid the obstacles safely and have completed the pursuit of the target UAVs when the formation target UAVs escape to the coordinates (170, 170), and then the task UAVs adjust the formation to achieve a better encirclement of the target UAVs. In multiple simulation comparisons, the success rate of the UAV’s adjusted formation rounding up increased from the initial 60% to more than 90% compared to the formation rounding up before the adjustment. The path of the roundup process is smoothed using Bessel curves.

**Figure 11:**
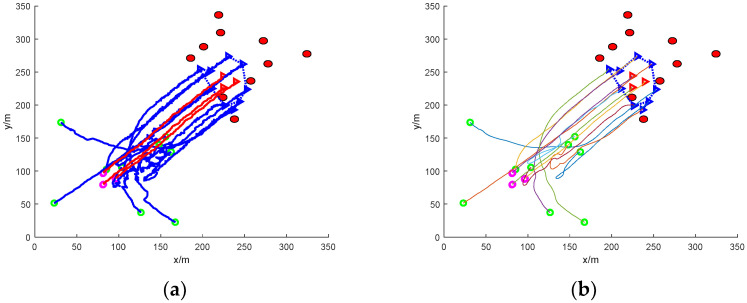
Nine UAVs rounding up three formation dynamic targets under dynamic obstacles. (**a**) The roundup process. (**b**) Smoothing of the roundup process.

Figure 11 shows the simulation of nine UAVs rounding up three formation dynamic targets under dynamic obstacles. The dynamic obstacles are near the target the UAVs, and the mission UAVs maintain a safe distance from the dynamic obstacles during the process of rounding up the target UAVs and complete the rounding up. Finally, the path of the roundup process is smoothed to obtain a more stable roundup path. In many simulation comparison experiments, although the UAV path before smoothing can also complete the roundup, the success rate of the UAV’s formation is not good, and often results in formation failure, with an average failure rate as high as 20%, while the formation failure rate of the UAV’s roundup path after the smoothing treatment is only 2%, and compared with before and after the smoothing, the success rate of the UAV’s formation in the process of roundup increases by more than 10 times.

**Figure 12:**
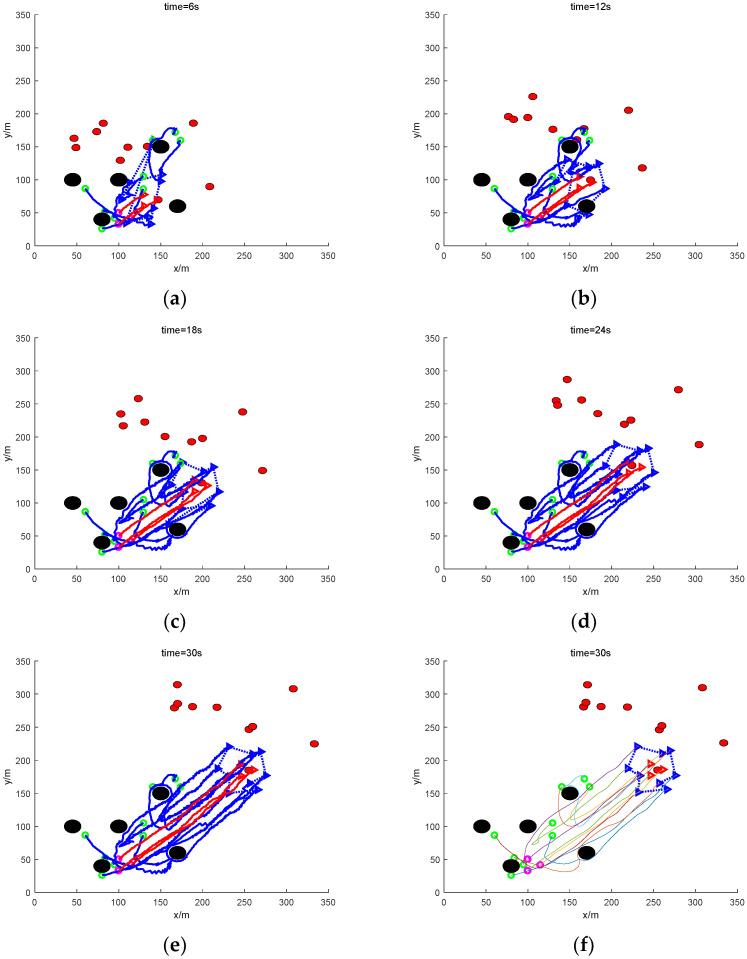
Sampling diagram of the process of nine UAVs rounding up three formation dynamic targets under mixed obstacles. (**a**) The roundup process (6 s moment). (**b**) The roundup process (12 s moment). (**c**) The roundup process (18 s moment). (**d**) The roundup process (24 s moment). (**e**) The roundup process (30 s moment). (**f**) Smoothing of the roundup process (30 s moment).

In Figure 12, the results of the traditional method of rounding up at 6 s, 12 s, 18 s, 24 s, and 30 s are shown in order, respectively. For the target UAVs with the formation, the roundup can be completed because the roundup target does not need to be distinguished and does not involve the problem of allocation. At 6 s, the task UAVs converge to the target UAVs; at 12 s, a preliminary roundup has been achieved; at 18 s, the roundup is completed; and from 24 s up to the end of the 30 s simulation, the roundup formation of the UAVs basically remains unchanged. It shows that the real-time performance of roundup is good. However, since the traditional method fails to round up targets that are scattered or escaping in groups, the real-time performance of rounding up is meaningless. The improved method is able to complete the rounding up and also ensure good real-time performance.

**Figure 13:**
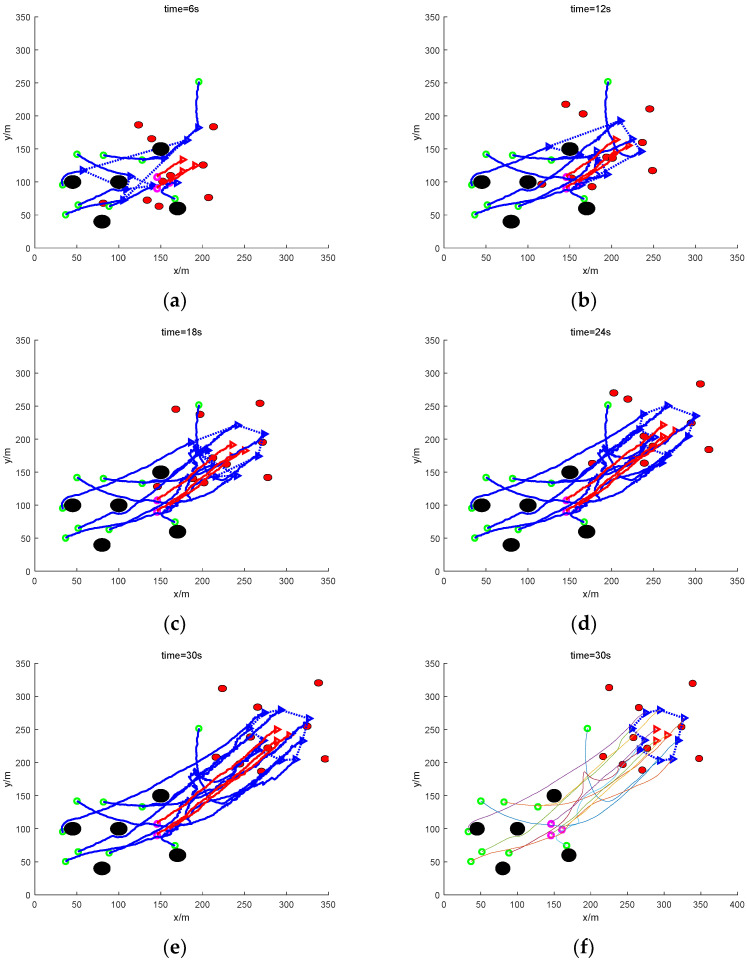
Sampling diagram of the process of nine UAVs rounding up three formation dynamic targets under mixed obstacles. (**a**) The roundup process (6 s moment). (**b**) The roundup process (12 s moment). (**c**) The roundup process (18 s moment). (**d**) The roundup process (24 s moment). (**e**) The roundup process (30 s moment). (**f**) Smoothing of the roundup process (30 s moment).

Figure 13 shows a simulation of nine UAVs rounding up three formation dynamic targets under mixed obstacles. In the figure, the static obstacles in Figure 8 and the dynamic obstacles in the case of Figure 9 are imposed. The mission UAV starts at the initial position of the green circle and avoids the black static obstacle while avoiding the red dynamic obstacle due to its presence. Then, by continuously converging towards the target UAV, it eventually forms an encirclement of the target UAV and achieves the roundup. The path of the encirclement process is smoothed.

In the figure, the rounding effect of this paper at 6 s, 12 s, 18 s, 24 s, and 30 s, respectively, is shown in order. It can be seen that, compared with the traditional method, the roundup effect of this paper’s method at each moment is relatively smoother. At 6 s, the mission UAV flew steadily towards the target UAV and formed a preliminary roundup at 12 s. The UAV completed the roundup at 18 s. And then, after 24 s, and finally at the end of the 30 s simulation, the UAV’s roundup formation basically remained the same, and the path of roundup was relatively stable. The real-time performance of the roundup is guaranteed.

(3) Escape in groups: nine mission UAVs are split into two groups and work together to round up three escaped targets

**Figure 14:**
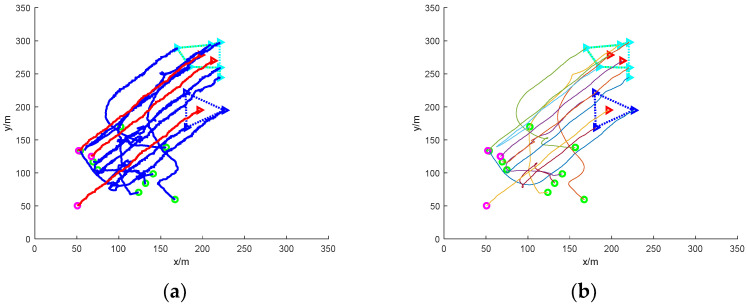
Nine UAVs rounding up grouped escaping dynamic targets under no obstacles. (**a**) The roundup process. (**b**) Smoothing of the roundup process.

Figure 14 is a simulation diagram of nine UAVs rounding up a dynamic target escaping in groups with no obstacles. In the figure, the target UAVs are divided into two groups to escape: one group consists of two target UAVs escaping in formation, and the other group consists of one target UAV escaping alone. Then, the mission UAVs need to distribute the UAVs and change the formation according to the actual situation. It can be found that the task UAV randomly distributes the initial position, detects the existence of target UAVs in the environment in two formations and one alone, so the task UAV matches the corresponding number of UAVs according to the actual distance and the number of target UAVs to pursue the target UAVs, and in the process of pursuing the target UAVs, changes the formation to realize the encirclement of the target UAVs. Then, the flight path of the UAVs is smoothed to obtain a stable encirclement path.

**Figure 15:**
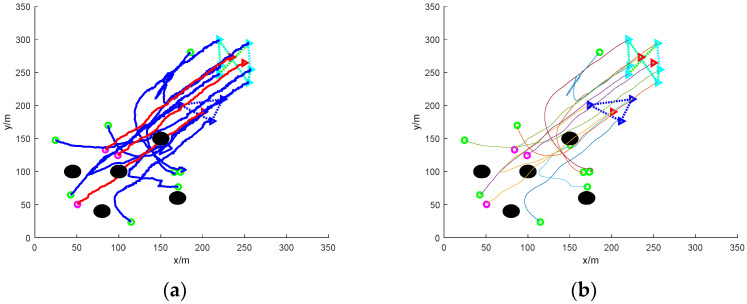
Nine UAVs rounding up grouped escaping dynamic targets under static obstacles. (**a**) The roundup process. (**b**) Smoothing of the roundup process.

Figure 15 shows the simulation diagram of nine UAVs rounding up a dynamic target escaping in a group under static obstacles. Due to the existence of static obstacles, in order to ensure the safety of the mission UAVs, the static obstacles are avoided, and then, according to the principle of linear matching, the number of mission UAVs in the assigned formation is used to encircle the target UAVs. The final roundup is successful, and the flight path of the roundup process is smoothed to obtain a smooth flight path without collision.

**Figure 16:**
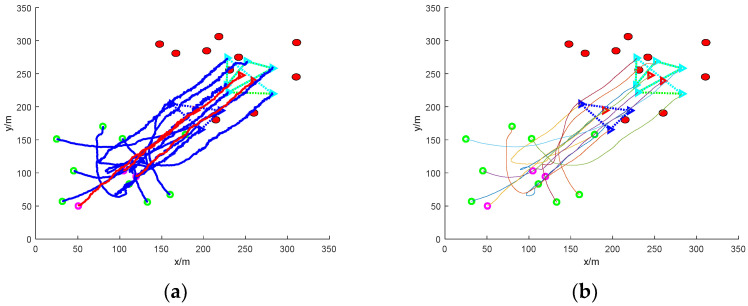
Nine UAVs rounding up grouped escaping dynamic targets under dynamic obstacles. (**a**) The roundup process. (**b**) Smoothing of the roundup process.

Figure 16 shows the simulation diagram of nine UAVs rounding up grouped escaping dynamic targets under dynamic obstacles. Due to the real-time movement of the dynamic obstacles, which causes some obstacles for the task UAV’s rounding up, the task UAV achieves the rounding up of the target UAVs under the condition of ensuring safety. The flight path of the roundup process is smoothed.

**Figure 17:**
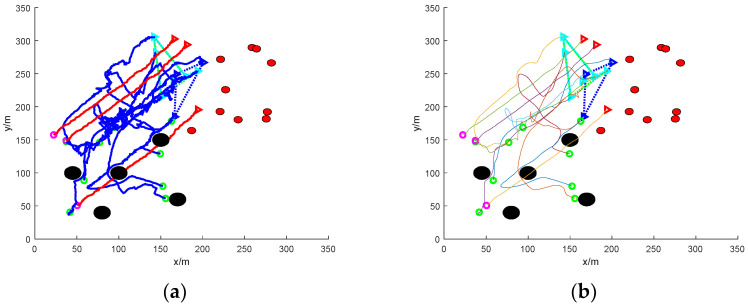

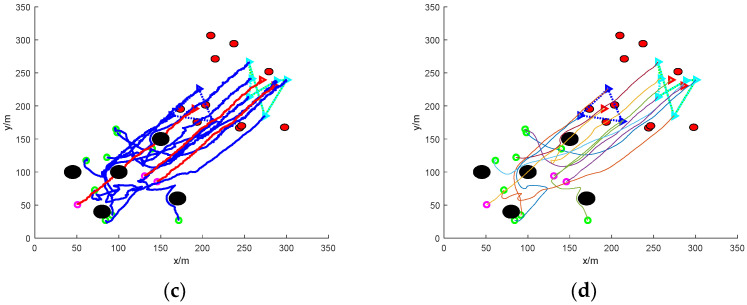
Nine UAVs rounding up grouped escaping dynamic targets under mixed obstacles. (**a**) Traditional methods of roundup process. (**b**) Smooth traditional method roundup process. (**c**) The roundup process of this paper’s method. (**d**) Smoothing of the roundup process of the method in this paper.

Figure 17 shows the simulation of nine UAVs rounding up grouped escaping dynamic targets under mixed obstacles. In the figure, namely, there are static obstacles and dynamic obstacles. In this extreme case, in order to ensure the safety of the mission UAVs, the static obstacles are evaded while some dynamic obstacles hinder the flight and need to be circumvented and evaded. At the same time, the corresponding number and proximity of the mission UAVs are assigned in real time to round up the target UAVs. Finally, the encirclement is completed, and the UAV flight path is smoothed using the Bessel curve approach to obtain a smooth collision-free encirclement path.

In the figure, the traditional method fails to achieve rounding up while the method in this paper succeeds in rounding up. Figure 17a is the roundup process of the traditional method, and Figure 17b is the smoothing process of the roundup process of the traditional method, and it can be clearly found that the traditional method’s roundup course in the interval from [50, 130] to [120, 200] is frequently changed in reverse, which illustrates that some of the mission UAVs switch the roundup target frequently, and then the formation in which they are located is also uncertain. Eventually, it affects the whole system and leads to mission UAV roundup failure.

Figure 17c shows the rounding up process of the proposed method, in which the UAV has finished rounding up one group of escaped targets at the position of (150, 150) coordinates, and then the flight path remains smooth until the end of the simulation at the coordinates of (200, 200). And the rounding up of another group of targets is also completed at (165, 135) coordinates until the end of the simulation, and the completion of rounding up remains basically unchanged. Figure 17d shows the smoothing process of the rounding process of this paper’s method, and the smoothed path is more stable. In practice, using the smoothed path causes a certain improvement on the stability and success rate of the roundup.

## 5. Conclusions

This research project reviews UAV roundup and obstacle avoidance studies. After demonstrating UAV group coordination and target escape methods, this paper proposes a real-time multi-UAV roundup algorithm for multiple dynamic targets. Unlike traditional methods, the proposed method can not only round up a single formation escaping target in real time, but also round up multiple targets scattered and grouped together, which solves the problem of rounding up failures due to the distribution problem of traditional methods.

In this study, the UAV performing the roundup mission is placed in environments with no obstacles, static obstacles only, dynamic obstacles only, and mixed obstacles, and the target UAVs in the scattered escape, formation escape, and group escape situations are rounded up, respectively. Simulation analyses were conducted one by one to verify the correctness of the algorithm. The results show that the rounding up efficiency of the formation is greatly improved, resulting in a 50% increase in the rounding up efficiency and an improvement in the success rate of the formation by more than 10 times. The success rate of this paper’s method in rounding up formation targets is increased from 60% to more than 90% compared with the traditional method. The success rate of the traditional method in rounding up dispersed escaping targets and group escaping targets is less than 2%, while the method in this paper has been simulated many times, and the success rate can reach more than 90%. The flight path of the UAV is smoothed by using Bessel curves to obtain a smooth and steady flight path. It provides a certain reference value for the application related to multi-target rounding up.

Nevertheless, there are still some problems worth thinking about and paying attention to and expanding upon in further research, including the study of the practice and optimization of the algorithm. The algorithm proposed in this paper has some limitations and potential challenges. The main limitation is that when dealing with a situation where the number of target UAVs is larger than that of the mission UAVs, rounding up is difficult and there will be some escaped target UAVs with no way for the mission UAVs to round them up, which deserves further research. And the potential challenges come from factors such as electromagnetic interference in real-life environments, which requires an improvement in the ability of perturbation suppression. Moreover, UAV localization and sensing is challenging due to the presence of errors in the sensors.

## Figures and Tables

**Table 1:** Number and radii of UAVs and obstacles in the environment versus speed.

	Mission UAV	Target UAV	Static Obstacles	Dynamic Obstacles
Number (pcs)	9	3	5	10
Radius (m)	1	1	10	5
Speed (m/s)	5~20	5~10	0	−10~20

**Table 2:** Datasheets for UAV components and modules and channel parameters.

No.	Component Name	Parameter Setting	Parameter Data
1	Main control unit	Preset flight modes, automatic stabilization control	Processor: STM32F427, frequency: 168 MHz
2	GPS module	High-precision positioning mode	Positioning accuracy: 2 m, update rate: 10 Hz
3	IMU sensor	Integrated accelerometer and gyroscope	Accelerometer range: ±40 g, gyroscope range: ±2000°/s
4	Communication module	Frequency setting, power adjustment	Frequency range: 902–928 MHz, transmission distance: 9 km
5	Drive module push	Thrust and RPM settings	Thrust: 2200 g, speed: 2400 rpm
6	Drive module	Thrust and speed settings	Thrust: 2200 g, speed: 2400 rpm
7	Power module	Voltage monitoring, discharge rate	Capacity: 5000 mAh, discharge rate: 20 C
8	Camera module	Resolution and frame rate settings	Resolution: 1280 × 960, frame rate: 60 fps

**Table 3:** Parameter setting support and data sheets.

Parameter Setting	Parameter Data	Parameter Setting	Parameter Data
Constant coefficient ($\mu_{1}$)	3	Obstacle point repulsion coefficient (β)	100
Exponential constant coefficient (λ)	2	Maximum distance of obstacle point repulsion ($D_{\max}$)	14 m
Boundary value of repulsion region ($R_{1}$)	10 m	Boundary value of target UAV escape state (R)	10 m
Equilibrium region boundary value ($R_{2}$)	30 m	Weight value constant ($\omega_{1}$)	1
Equilibrium region boundary value ($R_{3}$)	100 m	Weight value constant ($\omega_{2}$)	1
Constant coefficient ($\mu_{2}$)	2	Weight value constant ($\omega_{3}$)	1
Interference coefficient of the angle of motion	0.1		

## Data Availability

All data generated or analyzed in this study are included in the manuscript. All data in this study are available upon request from the corresponding author.
